# Granuloma Annulare in the Setting of Secukinumab

**DOI:** 10.1155/2018/5175319

**Published:** 2018-04-01

**Authors:** Matthew L. Clark, Courtney A. Tobin, Angela Sutton, Tricia Ann Missall

**Affiliations:** ^1^Department of Dermatology, Saint Louis University, 1755 S. Grand Blvd., Saint Louis, MO 63104, USA; ^2^Distinctive Dermatology, 510 Fullerton Rd., Swansea, IL 62226, USA

## Abstract

Granuloma annulare (GA) is a common benign inflammatory skin condition classically presenting as skin-colored to erythematous dermal papules and annular plaques. Histologically, GA displays a dermal granulomatous infiltrate with palisaded histiocytes surrounding focally altered collagen. The exactly etiology of GA remains unknown, but it has been associated with trauma, various infections, diabetes mellitus, malignancy, thyroid disease, dyslipidemia, and several medications. In 2017, a case of GA developing in a patient treated with the interleukin 17A antagonist secukinumab was reported. Here we report a second case of GA in association with secukinumab use.

## 1. Introduction

Granuloma annulare (GA) is a common benign inflammatory skin condition classically presenting as skin-colored to erythematous dermal papules and annular plaques. The exactly etiology of GA remains unknown, but it has been associated with trauma, various infections, diabetes mellitus, malignancy, thyroid disease, dyslipidemia, and several medications [1]. Here we report a second case of GA developing in a patient treated with the interleukin 17A antagonist secukinumab.

## 2. Case Report

**Figure 1:**
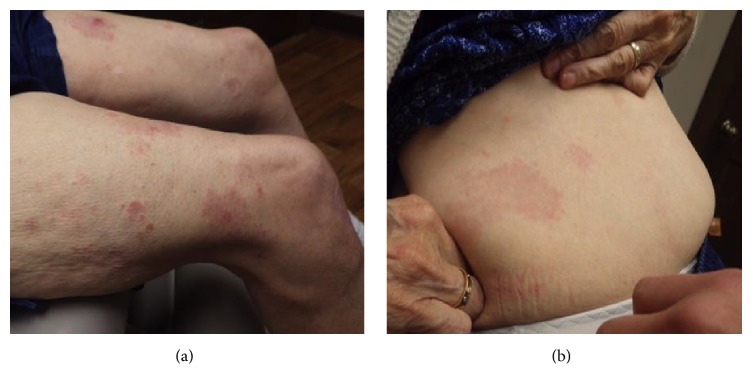
Patient's presentation with a new eruption in June 2017.

**Figure 2:**
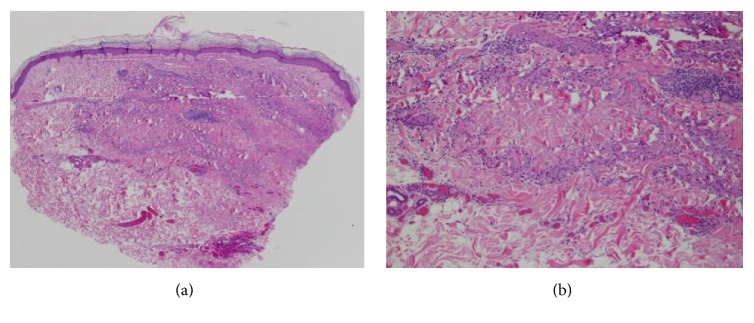
Palisaded histiocytes between focally altered collagen consistent with GA.

A 69-year-old Caucasian female with past medical history of hypertension, diabetes mellitus (DM), hyperlipidemia (HLD), and anxiety, initially presented in 2014 for treatment of psoriasis. Her home medications included lisinopril, metformin, pravastatin, citalopram, and alprazolam. Physical exam revealed greater than 20 percent body surface area affected by pink scaly plaques consistent with psoriasis. She was started on adalimumab and topical steroids and initially responded well. In June of 2016 her psoriasis was no longer well controlled on adalimumab alone, and apremilast was added to her treatment regimen. The patient experienced an unsatisfactory response to this combination therapy, with approximately five percent body surface area still affected. In January 2017, both medications were stopped and she was started on secukinumab. In June of 2017 she noted a new nonpruritic eruption on her legs and lower abdomen (Figures 1(a) and 1(b)). Physical exam revealed multiple discrete mauve dermal papules and annular plaques distributed primarily over her trunk and proximal lower extremities. A punch biopsy was performed, which revealed a dermal granulomatous infiltrate, composed of palisaded histiocytes surrounding focally altered collagen (Figures 2(a) and 2(b)), consistent with a diagnosis of GA. Once histologic evidence of GA was confirmed, secukinumab was discontinued in July 2017. The patient was treated for three months with triple antibiotic therapy with rifampin, levofloxacin, and minocycline. In September 2017, the eruption was still present and the patient was started on etanercept. At six-week follow-up, the GA had completely resolved.

## 3. Discussion

GA has been associated with several medications including allopurinol, amlodipine, diclofenac, gold, intranasal calcitonin, and TNF-*α* inhibitors, as well as other concomitant medical disorders including DM, HLD, and thyroid disease [1]. Our patient does have a history of DM (glycosylated hemoglobin 7.0 in September 2017) and HLD, both of which have been reported in association with GA. However, these conditions in our patient were overall stable and relatively well controlled, and conflicting evidence exists regarding the nature of the connection between these conditions and GA [2–4]. Given the temporal relationship of the development of her symptoms with the start of secukinumab, this is thought more likely the inciting event.

Cases of GA developing in patients being treated with anti-tumor necrosis factor-*α* (anti-TNF-*α*) medications have previously been reported. In 2007, Voulgari et al. reported nine cases of GA out of 199 patients (4.5%) being treated with anti-TNF-*α* therapy for rheumatoid arthritis and spondyloarthropathies. Of the nine patients who developed GA, six were treated with adalimumab, two were treated with infliximab, and one was treated with etanercept [5]. Of note, recalcitrant GA has also been shown to respond to treatment with several anti-TNF-*α* inhibitors [6, 7].

The exact pathomechanism of GA is yet to be fully elucidated. Increased expression of TNF-*α* and the matrix metalloproteinases MPP2 and MMP9 by activated macrophages is thought to play a role in the development of GA [8]. Additionally, increased interleukin 2 expression in biopsies of GA supports a T-helper 1 cell-mediated process [9]. The interaction between T-helper 1 and T-helper 17 cells is complex and studies have shown that they both cooperate and counter-regulate each other [10]. As such, it is possible that interleukin 17 blockade may contribute to a shift to the T-helper 1 cell-mediated development of GA. Interestingly, while interleukins 12 and 23 are known to be upstream promoters of T-helper 17 cells, no cases of GA development in patients being treated with the interleukin 12/23 antagonist ustekinumab have been reported.

In 2017, Bonomo et al. reported the first case of a patient developing GA while being treated with the interleukin 17A antagonist secukinumab [11]. Here we report a second case of a patient developing GA while on secukinumab. While it is difficult to draw direct conclusions that secukinumab may increase the risk of developing GA, clinicians should be aware of this potential association.

## Abbreviations

anti-TNF-*α*: Anti-tumor necrosis factor-*α*
DM: Diabetes mellitus
GA: Granuloma annulare
HLD: Hyperlipidemia.
